# Editorial: New frontiers in pancreatic cancer care: Multidisciplinary approaches, the role of Pancreas Units, and their organizational impacts

**DOI:** 10.3389/fsurg.2023.1182206

**Published:** 2023-03-23

**Authors:** Lorenzo Cobianchi, Francesca Dal Mas, Stefano Denicolai, Pietro Previtali, Alessandro Venturi

**Affiliations:** ^1^ Department of Clinical, Diagnostic and Pediatric Sciences, University of Pavia, Pavia, Italy; ^2^ Department of General Surgery, IRCCS Policlinico San Matteo Foundation, Pavia, Italy; ^3^ ITIR – Institute for Transformative Innovation Research, University of Pavia, Pavia, Italy; ^4^ Department of Management, Ca’ Foscari University, Venice, Italy; ^5^ Department of Economics and Management, University of Pavia, Pavia, Italy; ^6^ Department of Political and Social Sciences, University of Pavia, Pavia, Italy; ^7^ Bureau of the Presidency, IRCCS Policlinico San Matteo Foundation, Pavia, Italy

**Keywords:** pancreatic cancer, Pancreas Units, organizational culture, change management, multidisciplinarity, clinical outcome, patient – centered care

**Editorial on the Research Topic** New frontiers in pancreatic cancer care: Multidisciplinary approaches, the role of pancreas units, and their organizational impacts

Pancreatic cancer stands as one of the most dangerous oncological diseases, with low survival rates (1, 2). The biological and treatment complexity of pancreatic cancer calls for a multidisciplinary and integrated approach to diagnosing and caring for the disease, involving different highly-specialized medical professionals from the fields of oncology, surgery, radiotherapy, endoscopy, and radiology, among others. In addressing this challenge, several novel integrated protocols combining chemotherapy, radiation treatment, and surgery are currently under development globally (3–5), and so are e-health and telemedicine solutions tailored explicitly for pancreatic cancer patients (6, 7).

Still, working on the clinical side is not enough to ensure pancreatic cancer patients with a patient-centric model of care, especially considering the multidisciplinary efforts needed and the complexity of the disease. One organizational response seems to come from the development and establishment of the Pancreas Units, which are brand-new organizational structures where professionals from several disciplines are present (8, 9). The patient's journey in a Pancreas Unit should allow the matching of several medical specialities, whose professionals should contribute in an integrated and predefined way to patient care and research. A similar concept of “Disease Units” has been successfully applied in other oncological fields, primarily through establishing Breast Units, which recorded higher survival rates and quality of life than non-specialized facilities (10).

Pancreas Units should lead to an effective response to pancreatic cancer patients’ clinical needs. Still, their multidisciplinary approach and the need for different professionals to join forces in a systematic way require addressing several organizational issues. Such themes include the knowledge management and translation processes and facilitators to allow the effective transfer, sharing, and creation of new knowledge to provide patients with the best possible care, nourishing, at the same time, datasets about the disease and its dynamics.

The development and establishment of such Pancreas Units have already started in several countries (8, 9), but the debate on how such Units should work is still open.

Starting from these premises, the purpose of the research topic was to gather the most recent developments and undergoing studies in pancreatic cancer care following a multidisciplinary approach, including their organizational, managerial, and healthcare implications, their impacts on surgical and oncological practice, and clinical decision-making.

The special topic gathered five contributions: one literature review, one empirical study, one opinion paper, one retrospective study, and one observation study.

The paper by Chen et al. aimed to compare the safety and overall effect of two surgical techniques, namely, robotic distal pancreatectomy and laparoscopic distal pancreatectomy, after the learning curve, especially in the perioperative outcome and short-term oncological outcome. To achieve this goal, the authors conducted a literature review, including 15 eligible articles. The authors’ findings reveal that there seem to be no significant differences between the two techniques, considering several aspects, namely the operative time, overall complications, major complications, blood loss, blood transfusion, reoperation, readmission, postoperative pancreatic fistula, and lymph node dissection.

The empirical study by Charalampopoulou et al. aimed to investigate whether the presence of factors released by irradiated fibroblasts affected the migratory and invasive capacity of pancreatic cancer cells exposed to different doses of photons or carbon ion radiotherapy. Results underlined how irradiated fibroblasts affected the invasiveness capability of pancreatic cancer cells, probably due to the reciprocal release of soluble factors. Findings call for more in-depth multidisciplinary studies.

In their opinion article, the research group of Herremans et al. reflected on the opportunities brought forth by precision medicine in overcoming disparities through the promotion of patient inclusion and provider diversity.

Quero et al. aimed to study the potential impact of the multidisciplinary tumour board (MDTB) on pancreatic cancer management by analyzing how the MDTB may influence pancreatic cancer diagnosis and treatment, with a particular focus on resectability assessment and the correspondence between MDTB definition of resectability and intraoperative findings. The 487 cases included in the analysis underlined a high concordance rate between MDTB resectability definition and intraoperative findings.

Last but not least, the observation study by Han et al. conducted in China aimed to shed light on the influence of the COVID-19 Omicron Variant on pancreatic cancer. Results, even on a small patient sample, highlighted the poor outcomes of COVID-19 in pancreatic cancer patients, calling for protective measures to protect such vulnerable patients.

The contributions to the research topic “New Frontiers in Pancreatic Cancer Care: Multidisciplinary Approaches, the role of Pancreas Units, and their Organizational Impacts” allowed us to study pancreatic cancer care through different lenses. Some takeaways emerge.

First of all, the accepted articles stressed the fundamental role of multidisciplinarity in dealing with such a complex disease. The synergy among chemotherapy, surgery, and radiotherapy, even with particles, represents a winning strategy in which outcomes, patients’ eligibility, and facilitating conditions must be measured and assessed (2). The role of MDTB and other multidisciplinary committees looks fundamental to enabling clinical decision-making. Tools should be put in place to ensure safety and patients’ management with an interdisciplinary approach, even more, following complex scenarios like the one provided by the COVID-19 emergency.

Second, more research is needed to investigate how to provide pancreatic cancer patients with the best possible outcomes. Applying different surgical techniques may lead to similar clinical results. Still, there seems to be room for further investigation. Precision medicine may represent a new frontier to improve equity in access to care. Again, the chemotherapy/surgery/radiotherapy synergy may offer the opportunity to design new experimental trials.

Last but not least, when multidisciplinarity and new frontiers in research are in place, organizational and managerial aspects appear crucial to coordinate the various stakeholders’ efforts. One of the key roles of the future Pancreas Units will be to facilitate the multiple professionals from the clinical and research world to develop and share their knowledge while, at the same time, offering to the patient and the family a precise and fruitful care path.

The debate about the strategic role of Pancreas Units, the way they should be managed, and the multistakeholder engagement they should foster has just getting started, and it does represent a cutting-edge topic in fighting one of the most challenging diseases in the oncological scenario.
